# Concordance between Immunohistochemistry and Microarray Gene Expression Profiling for Estrogen Receptor, Progesterone Receptor, and HER2 Receptor Statuses in Breast Cancer Patients in Lebanon

**DOI:** 10.1155/2018/8530318

**Published:** 2018-05-31

**Authors:** Ghina B. Fakhri, Reem S. Akel, Maya K. Khalil, Deborah A. Mukherji, Fouad I. Boulos, Arafat H. Tfayli

**Affiliations:** ^1^Division of Hematology and Oncology, Department of Internal Medicine, American University of Beirut, Beirut, Lebanon; ^2^Roswell Park Comprehensive Cancer Center/ State University at Buffalo-Jacobs School of Medicine and Biomedical Sciences, Elm & Carlton Streets, Buffalo, NY 14263, USA; ^3^Department of Pathology and Laboratory Medicine, American University of Beirut, Beirut, Lebanon

## Abstract

**Introduction:**

Accurate evaluation of estrogen and progesterone receptors and HER2 is critical when diagnosing invasive breast cancer for optimal treatment. The current evaluation method is via immunohistochemistry (IHC). In this paper, we compared results of ER, PR, and HER2 from microarray gene expression to IHC in 81 fresh breast cancer specimens.

**Methods:**

Gene expression profiling was performed using the GeneChip Human Genome U133 Plus 2.0 arrays (Affymetrix Inc). Immunohistochemical staining for estrogen receptor, progesterone receptor, and HER2 status was performed using standard methods at a CAP-accredited pathology laboratory. Concordance rates, agreement measures, and kappa scores were calculated for both methods.

**Results:**

For ER, Kappa score was 0.918 (95% CI, 0.77.3–1.000) and concordance rate was 97.5% (95% CI, 91.4%–99.7%). For PR, Kappa score was 0.652 (95% CI, 0.405–0.849) and concordance rate was 86.4% (95% CI, 77%–93%). For HER2, Kappa score was 0.709 (95% CI, 0.428–0.916) and concordance rate was 97.5% (95% CI, 91.4%–99.7%).

**Conclusion:**

Our results are in line with the available evidence with the concordance rate being the lowest for the progesterone receptor. In general, microarray gene expression and IHC proved to have high concordance rates. Several factors can increase the discordance rate such as differences in sample processing.

## 1. Introduction

Breast cancer is the most common cancer in women. Several tumor characteristics play a major role in determining optimal management of this tumor. Estrogen receptor (ER), progesterone receptor (PR), and HER2-neu have emerged as critical prognostic and therapeutic markers for diagnosis and treatment [1–5]. For optimal treatment, all breast carcinomas are tested for these three markers. The current standard of care for testing is via immunohistochemistry (IHC) alongside Fluorescent In Situ Hybridization (FISH) for HER2 when equivocal. However, several studies have shed light on the discordance rates and interobserver agreement in scoring hormone receptor status which are mostly related to effects of fixation, choice of antibody, and scoring interpretation [6–13]. The authors of one article [14] found that up to 20% of the IHC test results of ER and PR statuses could be inaccurate while other articles have found that the false-negative and false-positive rates can reach numbers much higher than 20% [15, 16]. To address the discordance rates and other limitations set by IHC, several studies have been performed to accurately determine hormone receptor status using gene analysis. Because information on hormone receptor status determines eligibility for different treatment modalities, more data have been emerging on the use of microarray genetic testing to further improve the accuracy of PR, ER, and HER2 test results. Most studies have shown high concordance rates between IHC ER, PR, and HER2 results and microarray gene expression profiling paving the way for the use of the latter solely for classification of breast carcinomas [17–21]. In this study, we compare the results of ER, PR, and HER2 gene expression via microarray analysis to those from IHC in 81 fresh breast cancer tissue samples.

## 2. Materials and Methods

### 2.1. Subjects and Samples

This research study was approved by the Institutional Review Board (IRB) and all subjects provided written informed consent. The study was conducted in accordance with the precepts established by the Helsinki Declaration. 81 fresh tissue specimens were collected from females who were newly diagnosed with stage I, II, or III breast cancer between September 2012 and May 2014. Tumor cells were assessed histologically to confirm a diagnosis of invasive ductal carcinoma and/or invasive lobular carcinoma and to ensure the presence of sufficient tumor cells.

### 2.2. Microarray Gene Expression

Extraction of unique mRNA fingerprints was performed using RNeasy® Plus Mini Kit (Qiagen). RNA samples were stored at −80°C. Their quality and yield were assessed using* A*_260_/*A*_280_ and* A*_260_/*A*_230_ and ratios were analyzed with Experion™ Automated Electrophoresis System (Biorad). RNA concentrations were determined by absorption at 260 nm wavelength with a ND-1000 spectrometer (Nanodrop Technologies).

Gene expression profiling was performed using the GeneChip Human Genome U133 Plus 2.0 arrays (Affymetrix Inc.). 100 ng of RNA was amplified, labeled, fragmented, and hybridized using GeneChip 3′ IVT Express Kit. Washing and staining were conducted using GeneChip Fluidics Station 450 and scanning of arrays was performed using a GeneChip Scanner 3000 7G. The Affymetrix GeneChip Command Console (AGCC) software (v3.2) generated data cell files (DTC). For the process of RNA amplification, labeling, and hybridization, details are available from the Affymetrix website (https://www.affymetrix.com). Microarray scores for ER, PR, and HER2 were considered positive if ≥0.

### 2.3. Quantitative Real-Time PCR

Confirmation of microarray results was performed by quantitative real-time PCR. Total RNA was reverse-transcribed using RevertAid Reverse Transcriptase (Thermo Scientific) with 100–1000 ng of input RNA and random primers (Thermo Scientific). Quantitative real-time PCR reactions were performed in 96-well plates using specific primers (TIB MOLBIOL) and the iQTM SYBR® Green Supermix (BioRad) as a fluorescent detection dye, in CFX96TM Real-Time PCR (BioRad), in a final volume of 12.5 *μ*l. To characterize generated amplicons and to control contamination by unspecific by-products, melt-curve analysis was applied. Each reaction was performed in duplicate. All results were normalized to PGK1 mRNA level and calculated using the ΔΔCt method. The specificity of the PCR was determined by melt-curve analysis for each reaction.

### 2.4. Immunohistochemical Staining

Fresh tumor tissue sections were fixed with formalin and embedded in paraffin. Immunohistochemistry staining of ER, PR, and HER2 markers was performed at a College of American Pathologists- (CAP-) accredited Pathology Department. For antigenic retrieval citrate buffer (pH 6) was used for the ER antibody and EDTA (pH 8) was used for the PR antibody. Immunostains were performed using a polymer detection system. ER and PR scores were determined based on the percentage of tumor cells showing positive nuclear staining and were considered positive if nuclear staining was present in ≥1% of the cells according to the ASCO/CAP guidelines [22]. For the HER2 status, scoring is as follows: 0 for no staining, or membrane staining in less than 10% of tumor cells, 1+ for a faint membrane staining in ≥10% of tumor cells, 2+ for weak to moderate membrane staining in ≥10% of tumor cells, and 3+ for strong membrane staining in ≥10% of tumor cells. ER and PR are considered positive if there is membrane staining in more than 1% of tumor cells. HER2 overexpression is considered negative if HER2 score is 0 or 1+, and positive if HER2 score is 3+. Tumor cells with a HER2 score of 2+ were further evaluated using Fluorescence In Situ Hybridization (FISH). The aforementioned procedures are Food and Drug Administration- (FDA-) approved.

### 2.5. Estrogen Receptor and Progesterone Receptor

ER staining was performed using Invitrogen 1D5 clone and PR staining was performed using Invitrogen PR-2C5 clone. Positive and negative controls were included in each slide run and all controls showed appropriate reactivity.

### 2.6. HER2 Status

Immunostaining for HER2 was performed using Dako polyclonal. Positive and negative controls, included in each slide run, showed appropriate reactivity. Determination of HER2 overexpression status was performed using HercepTest scoring system. When required, FISH was performed using Pathvysion HER2 DNA PROBE kit, with a probe specific for the Her2/neu gene locus (17q11.2-q12) and another probe specific for the centromeric region of chromosome 17 (CEP17) (17p11.1-q11.1). Samples evaluated for HER2 using FISH were considered positive when HER2/CEP17 > 1.80.

### 2.7. Clinical Covariables

In addition to ER, PR, and HER2 statuses obtained via IHC and microarray, other clinical covariables were obtained and used in the analysis and consisted of age at diagnosis, histologic type, histologic grade, size, and stage.

### 2.8. Statistical Analysis

Statistical analysis was performed using SPSS v24. Correlation of ER, PR, and HER2 between microarray analysis and immunohistochemistry was determined using measures of agreement. These measures included overall concordance, positive agreement (defined as the number of samples classified positive by both microarray and immunohistochemistry divided by the number of positive samples using immunohistochemistry), negative agreement, positive predictive value (PPV), negative predictive value (NPV), and Cohen's Kappa coefficient score (*κ*). Positive predictive value (PPV) is calculated as number of samples positive by both IHC and microarray divided by the number of samples positive via microarray analysis only. On the other hand, negative predictive value (NPV) is calculated as the number of samples negative by both IHC and microarray divided by the number of samples negative by microarray only. All measurements were associated with a confidence interval (CI) of 95% and all statistical tests were considered significant when *p* value < 0.05.

## 3. Results

The clinical and pathologic features of the 81 breast cancer tissue samples are summarized in Table 1.

Results of ER, PR, and HER2 statuses from immunohistochemistry and from gene expression profiling using microarray techniques were obtained and compared. Compared with immunohistochemistry, microarray results showed a concordance of 97.5% for ER, 86.4% for PR, and 97.5% for HER2. See Tables 2 and 3.

For ER, Kappa score was 0.918 (95% CI, 0.773–1.000), positive agreement was 100%, and negative agreement was 87.5%. All 65 samples scored positive by IHC were also positive for ER via microarray analysis whereas only 2 out of the 16 (12.5%) samples negative by IHC were positive via microarray analysis. The PPV and the NPV of ER are 97% and 100%, respectively. The discordance rate for ER is 2.5%.

For PR, Kappa score was 0.652 (95% CI, 0.405–0.849), positive agreement was 93.1%, and negative agreement was 69.6%. Only 4 out of the 58 (6.8%) samples positive by IHC were negative via microarray analysis. Out of the 23 samples negative by IHC, 7 were positive via microarray analysis (30.4%). The PPV and NPV are 88.5% and 80%, respectively, and the discordance rate is 13.6%.

For HER2, Kappa score was 0.861 (95% CI, 0.428–0.916), positive agreement was 77.8%, and negative agreement was 100%. Only 2 out of the 9 samples positive by IHC were negative via microarray (22.2%) whereas all samples positive by IHC were also positive via microarray analysis (100%). The PPV and NPV are 100% and 97.2%, respectively, and the discordance rate for HER2 was 2.5%.

No clear correlation was found between the agreement measures and patient clinical or histologic characteristics, most likely due to the small sample size.

## 4. Discussion

There is a growing need to accurately define the receptor status in patients with invasive breast cancer who are most likely to benefit from hormonal therapy. As many methods are arising to measure the statuses of ER, PR, and HER2 besides the long-practiced conventional immunohistochemistry, false positives and false negatives are also being reported. Several studies investigated the accuracy of alternative methods for these receptors in search for reliable and accurate results.

In our study, we found that the general results are in line with the available data. Many investigators used similar methodology comparing the status of these receptors to the standard IHC. Gong et al. [23] used Affymetrix U133A gene expression profiling and compared both ER and HER2 statuses to IHC. They reported 92% concordance rate for ER and 90% for HER2 which they believed to be both reproducible and reliable. Having used the same methodology in our study, we have reported even higher concordance.

Viale et al. compared TargetPrint microarray readouts to central IHC and results showed a concordance rate of 98% for ER, 92% for PR, and 75% for HER2, followed by secondary analysis for the discordant cases, and the overall conclusion was that the presence of DCIS or intratumoral heterogeneity does not support the reason for the discordance rates they previously reported [24, 25]. Viale also concluded that TargetPrint can improve the reliability of hormone receptor evaluation especially in centers with a lower rate of concordance. Wesseling et al. [20] also used the same TargetPrint modality in an attempt to compare ER, PR, and HER2 status to IHC and reported 95% concordance for ER, 81% for PR, and 94% for HER2. On the other hand, Roepman et al. [19] used Mammaprint (mRNA expression) modality and compared its results of ER, PR, and HER2 with those from IHC and its concordance rates were reported as 93% for ER, 83% for PR, and 96% for HER2.

Badve et al. [26] compared OncotypeDx (RT-PCR) to both local and central IHC for both ER and HER2 receptors. Comparison between central IHC and RT-PCR showed a concordance rate of 93% for ER and 90% for PR and they concluded that the degree of concordance between OncotypeDx and IHC, both local and central, is high. Another large case-control study lead by Baehner et al. [27] reported HER2 concordance rate between OncotypeDx and IHC as high as 97%.

In our study, the concordance rate was highest for ER status (97.5%) and lowest for PR hormonal status (86.4%) which is in line with other reported studies in the literature. The reason for this discordance in PR status is not clear and might be related to variation in the IHC testing methods used. It is well reported that the interobserver variability is highest in IHC staining for PR [19, 20].

For determining the positivity of ER and PR statuses in IHC, the cut-off for certain authors was set as 10% while others set it as 1% [15]. In our study, we have used the 1% cut-off and this generated a certain number of positive ER and PR which would change had the cut-off been increased to 10%. As such, the difference in the discordance rates can be explained by the different thresholds for receptor positivity used in different studies. Thus, the final decision as to which method to rely on and which method is more accurate and reliable remains to be determined in future prospective studies that can include both treatment type and response. Other authors have reported the discrepancies to be mostly due to the evaluation process, staining procedures, suboptimal sample processing, long-term fixation, and intratumoral heterogeneity [28, 29].

A limitation of our study is that the discordant cases were not reassessed by second pathologist or in a different central lab to assess for interrater reliability. A needed prospective research project would also be to compare ER and PR from adjacent normal breast especially in breast cancer that has been labeled as ER and/or PR negative. This can add accuracy and reliability to our microarray gene expression. Moreover, it is worth noting that IHC relies on protein expression while microarray readouts rely on mRNA expression and as such, differences between them are inherently expected. There is also uncertainty in both IHC and gene expression modalities as they both rely on the presence of protein or mRNA but none of these modalities truly determine whether these proteins are functional in themselves or whether their mRNA will produce functional proteins [19, 30] which opens the doors for future potential research in that field. Another difference that may account for discrepancies mentioned in earlier literature work is that microarray readouts were usually performed on fresh frozen tissue while IHC was performed on paraffin embedded tissue. This is less of an issue these days since microarray analyses can be done on FFPE [28, 29].

## 5. Conclusion

In conclusion, microarray readouts and IHC proved to have a high concordance rate as per our results and the aforementioned studies as well. Given the fact that IHC is more widely available, easier to do, and less costly, it is most reasonable to use IHC in everyday testing of breast cancer specimens. Microarray testing remains a reasonable back-up testing modality for quality control and other research purposes.

## Figures and Tables

**Table 1:** Clinical demographic variables.

Category	*N* (%)
Age (years)	
Mean, range	53, 29–84
Largest Diameter (cm)	
Mean, range	2.6 (0.7–7)
Stage	
I	34 (42)
II	36 (44.4)
III	9 (11.1)
IV	2 (2.5)
Histopathology type	
Invasive Ductal Carcinoma	69 (85.2)
Invasive Lobular Carcinoma	8 (9.9)
Mixed	1 (1.2)
Other	3 (3.7)
Grade	
1	25 (30.9)
2	29 (35.8)
3	27 (33.3)

**Table 2:** Microarray gene expression Values of ER, PR, and HER2 compared to Immunohistochemistry.

	IHC		
	Positive	Negative	Total *N* (%)
Microarray Analysis			
Microarray-ER			
Positive	65	2	67 (82.7)
Negative	0	14	14 (17.3)
Total *N* (%)	65 (80.2)	16 (19.8)	81
Microarray-PR			
Positive	54	7	61 (75.3)
Negative	4	16	20 (24.7)
Total *N* (%)	58 (71.6)	23 (28.4)	81
Microarray-HER2			
Positive	7	0	7 (8.6)
Negative	2	72	74 (91.4)
Total *N* (%)	9 (11.1)	72 (88.9)	81

**Table 3:** Concordance rates and agreement measures of microarray gene expression compared to Immunohistochemistry as reference.

	ER		PR		HER2	
	Value	95% CI	Value	95% CI	Value	95% CI
Concordance	0.975	0.914–0.997	0.864	0.770–0.930	0.975	0.914–0.997
Kappa	0.918	0.773–1.000	0.652	0.405–0.849	0.861	0.428–0.916
Positive Agreement	1.000	0.930–1.000	0.931	0.824–0.9777	0.778	0.401–0.960
Negative Agreement	0.875	0.604–0.978	0.696	0.469–0.859	1.000	0.936–1.000
Positive Predictive Value	0.970	0.886–0.994	0.885	0.771–0.948	1.000	0.560–1.000
Negative Predictive Value	1.000	0.732–1.000	0.800	0.557–0.933	0.972	0.896–0.995
